# Ultra-Wide-Field Optical Coherence Tomography Assessment of Choroidal Parameters in Central Serous Chorioretinopathy

**DOI:** 10.3390/diagnostics16131982

**Published:** 2026-06-25

**Authors:** Maciej Gawęcki, Karolina Mach, Andrzej Kwiatkowski, Krzysztof Kiciński, Jan Kucharczuk, Anna Święch, Dariusz Nałęcz, Andrzej Grzybowski

**Affiliations:** 1 Dobry Wzrok Ophthalmological Center, 80-392 Gdansk, Poland; karolina.mach@dobry-wzrok.pl (K.M.); andrzej.kwiatkowski@dobry-wzrok.pl (A.K.); krzysztofkg999@icloud.com (K.K.); 2 Department of Ophthalmology, Pomeranian Hospitals in Wejherowo, 84-100 Wejherowo, Poland; 3 Department of Ophthalmology, 10th Military Research Hospital and Polyclinic, 85-681 Bydgoszcz, Poland; jankucharczuk@wp.pl; 4 Department of Vitreoretinal Surgery, Medical University of Lublin, 20-081 Lublin, Poland; anna.swiech2022@gmail.com; 5 Department of Otolaryngology and Maxillofacial Surgery, St. Vincent De Paul Hospital, 81-348 Gdynia, Poland; dnalecz@szpitalepomorskie.eu; 6 Department of Ophthalmology, University of Warmia and Mazury, Oczapowskiego 2, 10-719 Olsztyn, Poland; ae.grzybowski@gmail.com; 7 Institute for Research in Ophthalmology, Foundation for Ophthalmology Development, Mickiewicza 24, 61-836 Poznan, Poland

**Keywords:** central serous chorioretinopathy, choroidal thickness, choroidal volume, ultra-wide-field OCT, pachychoroid, BCVA

## Abstract

**Purpose:** To analyze choroidal thickness (CT) and choroidal volume (CV) using ultra-wide-field (UWF) spectral-domain optical coherence tomography (SD-OCT) in patients with central serous chorioretinopathy (CSC) and to assess their associations with disease duration and best-corrected visual acuity (BCVA). **Methods:** This prospective case–controlled study included 50 eyes of 41 CSC patients and 56 eyes of 32 healthy controls matched for age and sex. CT was measured at 24 points using the REVO 130 UWF SD-OCT device with a wide-field adapter, covering a 21 × 21 mm retinal area across central, mid-peripheral (4 mm), and peripheral (8 mm) zones. CV was estimated using a quadratic nonlinear model. ROC curve analysis and univariate logistic regression were applied to evaluate discriminative capacity and odds ratios (OR) for CT and CV. **Results:** CT was significantly higher in CSC eyes at all 24 measurement points (all *p* < 0.0001). Mean subfoveal CT was 472.6 µm vs. 344.8 µm in controls (+37%), with greater relative increases at mid-peripheral (+46%) and peripheral (+44%) zones. Mean CV was 61.47 (±11.37) mm^3^ vs. 42.29 (±10.02) (+45%; *p* < 0.0001). CV showed a higher OR for CSC occurrence than central CT (OR = 2.88; 95% CI: 1.53–5.42 vs. OR = 1.04; 95% CI: 1.02–1.07). Significant discriminative CT points (AUC > 0.60) clustered at the 2/8, 4/10, and 6/12 clock meridians. Both CT and CV correlated positively with disease duration (Spearman rho 0.35–0.41; *p* ≤ 0.0004) but not with BCVA. **Conclusions:** UWF SD-OCT confirms diffuse pachychoroid thickening in CSC extending to the periphery. CV is a sensitive biomarker in association with CSC status. Peripheral CT and CV correlate with disease duration, supporting the link between higher volumetric choroidal values and longer disease course. Integration of these parameters may improve CSC diagnosis and prognostic evaluation.

## 1. Introduction

Central serous chorioretinopathy (CSC) is a common chorioretinal condition characterized by serous detachment of the neurosensory retina, most frequently affecting men in their fourth to sixth decades of life. Despite its prevalence, the pathogenesis of CSC remains incompletely understood, and treatment recommendations continue to evolve [1,2,3]. Early hypotheses centered on dysfunction of the retinal pigment epithelium (RPE) as the primary event; however, accumulating evidence has shifted focus toward abnormalities of the choroidal vasculature. In particular, the pachychoroid phenotype—characterized by dilation of the outer choroidal vessels (Haller layer), attenuation of the inner choroidal layers, and choroidal thickening—is now widely regarded as the structural substrate of CSC [1,2,3]. This concept has been further extended by the venous overload choroidopathy hypothesis, which proposes that impaired choroidal venous outflow leads to congestion and hyperpermeability, ultimately causing RPE breakdown and subretinal fluid accumulation [4].

In this framework, choroidal thickness (CT) has emerged as a key biomarker of CSC. Multiple studies and a large meta-analysis have demonstrated that subfoveal CT is substantially greater in CSC eyes than in healthy controls, and increased CT is now incorporated into the diagnostic criteria and characterization of the disease [5,6,7,8]. However, single-point subfoveal CT captures only a fraction of the choroidal architecture. Considering the choroid as a large, three-dimensional structure closely apposed to both the RPE and the sclera, a parameter describing its total volume—rather than thickness at a single point—more faithfully reflects its overall anatomy and disease burden. Estimation of choroidal volume (CV) requires imaging across a wide retinal area, which has become feasible with the advent of ultra-wide-field (UWF) optical coherence tomography (OCT) devices capable of measuring CT at both central and peripheral locations.

The aim of the present study was to assess CT and CV using UWF spectral-domain OCT (SD-OCT) in a cohort of CSC patients and to compare these parameters with those of healthy controls. We further sought to determine the discriminative capacity of peripheral CT measurements using Receiver Operating Characteristic (ROC) curve analysis and to evaluate correlations between choroidal parameters, disease duration, and best-corrected visual acuity (BCVA).

## 2. Material and Methods

All procedures performed in this study were conducted in accordance with the Declaration of Helsinki. The study protocol was approved by the local ethics committee Okręgowa Izba Lekarska (approval no. KB 40/24, dated 17 December 2024). Informed consent for all procedures performed during the disease management was obtained from all the participants of the study. The study group included 50 eyes of 41 consecutive individuals with CSC managed at Dobry Wzrok Ophthalmological Center between January 2025 and July of 2025. Control group included 56 eyes of 32 healthy consecutive individuals who underwent routine ophthalmological check-up for the occupational program within the same period of time as the study group. Control subjects were screened for past or present possible retinal or choroidal disorders with color fundus photography, fundus autofluorescence (FAF), and SD-OCT. Eyes with any abnormalities detected through these examinations or with a history of past or present ophthalmological disorders were excluded from the study. Patients with systemic conditions that could affect choroidal thickness (uncontrolled hypertension, disorders requiring corticosteroid use or obstructive sleep apnea) were excluded from the study. Cases with significant refraction error higher than −3.0 or +3.0 D in spherical equivalent were also excluded. Both study and control groups were matched for age and gender.

All of the patients of the study group presented with an active, non-resolved chronic type of disorder lasting longer than 6 months, with a median value of 35 months. Cases with recurrences of the activity of the disease were also included and regarded as chronic.

The CSC diagnosis criteria formulated by the International CSC Study Group were adopted [9]. These included past or present subretinal fluid (SRF) detected at SD-OCT and at least one pigmentary abnormality on FAF as primary criteria (both necessary). At least one of the three following secondary criteria was also present: leakage on fluorescein angiography or hyperpermeability of the choriocapillaris on ICGA. In our consecutive study cohort, all patients had a CT value > 400 μm. Duration of CSC was estimated on the basis of medical history and interview. The point of the first episode of CSC was considered the onset of the disease in recurrent cases. All of the study group cases received treatment with either classic photocoagulation or subthreshold micropulse laser (SML), but without the effect of permanent resolution of SRF. Patients who were treated with photodynamic therapy (PDT) were excluded due to the established PDT effect on choroidal thickness.

### 2.1. SD-OCT Measurements

**Figure 1:**
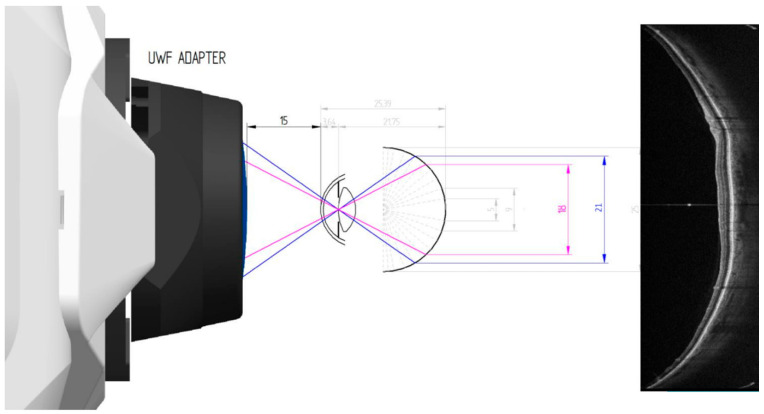
The diagram of the UWF adapter utilization for wide-field retina scanning.

SD-OCT measurements were performed using the REVO 130 device (REVO 130, 2023; Optopol Technology, Zawiercie, Poland) equipped with a UWF adapter developed by Optopol and enhanced depth imaging (New Enhanced Depth^TM^). The adapter enabled scanning of a retinal area measuring 21 × 21 mm. Acquisition of scans was scheduled in the morning between 8 and 12 AM. A schematic diagram of the device is presented in Figure 1.

**Figure 2:**
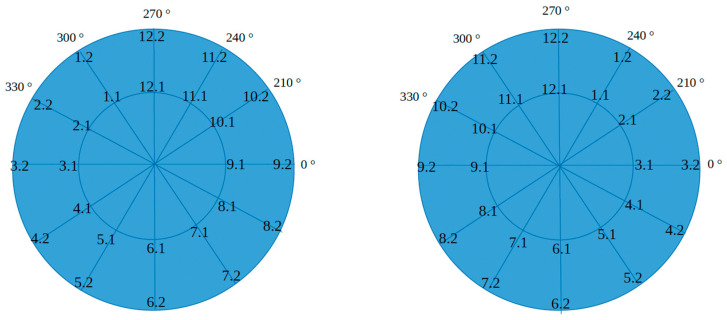
The diagram for the points of choroidal thickness measurements in the right and left eyes (right eye on the left and left eye on the right—facing the patient). The raster lines of measurement are located at 90, 120, 150, and 180 degrees.

Choroidal scans were flattened using software incorporated in the device. Subsequently, CT was measured manually using the built-in tools provided in the device software. Measurements were obtained at the central subfoveal point and points located on two concentric rings with radii of 4 mm and 8 mm (Figure 2). A raster scanning protocol was applied for this purpose, with four raster lines that translated into 12 measurement points at each circle. In the case of a 4 mm circle, 11 measurements were performed as one of the points was located directly at the optic nerve. Hence, altogether, measurements were taken in 23 points at the rings plus the central subfoveal (24 altogether). This applied to both the right and left eye. Mean and median values for subfoveal, medial (4 mm), and peripheral (8 mm) choroidal thickness were calculated. Two graders, one senior and one junior, performed the measurements. The measurements taken by the junior grader were subsequently verified by the senior grader. In cases of discrepancy of more than 15 μm, the final value was determined by inter-grader discussion.

### 2.2. Choroidal Volume Calculation

As it was described, choroidal thickness was measured at 24 points across the macular area, comprising a central measurement and 12 radial meridians (corresponding to clock-hour positions) sampled at 4 mm and 8 mm from the fovea. To account for meridian-related asymmetry in choroidal thickness, choroidal volume was estimated using a sector-based annular model rather than assuming radial symmetry. The scanned area (radius R = 8 mm) was divided into twelve 30° radial sectors corresponding to the clock-hour meridians. Within each sector, a quadratic radial thickness profile, h(r) = ar^2^ + br + c, was fitted independently using the central thickness h(0) and the meridian-specific thickness values at r = 4 mm and r = 8 mm. The volume of each sector was obtained by analytical integration of the solid-of-revolution formula restricted to the sector angle (θ = π/6 rad), and total choroidal volume was calculated as the sum of the twelve sector volumes. At the 9 o’clock meridian, where the r = 4 mm measurement was unavailable owing to its proximity to the optic disk, this value was estimated by linear interpolation between the central thickness and the corresponding r = 8 mm value.

Correlations between choroidal parameters and disease duration and BCVA were additionally assessed.

### 2.3. Statistical Analysis

Categorical variables were described through integer numbers and percentages (frequencies). Numerical variables were described through their mean, median, standard deviation, standard error, 95% confidence interval, and lower-to-upper quartile values. The normality of distribution was assessed by using the Shapiro–Wilk W test. Levene’s test was used to test the homogeneity of variances. A multifactor analysis of variance (ANOVA) without replications was performed in order to test the differences in normally distributed traits between the study groups. Due to the naturally binocular vision in the study subjects, specific intra-subject standard error correction methods were applied. Generalized linear models (GZM) with subject-level clustering were fitted (the clustered robust error correction), and Maximum Likelihood Estimation (MLE) procedures were fitted when dealing with non-normally distributed continuous variables and/or heteroscedastic variances. Analyzing dependent variables such as the study subjects’ age, thicknesses, and volume incorporated the Gaussian family and identity link function. The BCVA was handled by the gamma family, due to its very strong left-skewness, and log link function. The disease duration was handled by the gamma family, due to its right-skewness, and identity link function. Subsequent models were built by using a set of covariates: age, gender, and disease duration. False discovery rate correction has been applied for multiple hypothesis testing (*p* values, along with Simes’ q values were given). Spearman’s rank correlation coefficient was calculated when assessing relationships between non-normally distributed numerical variables. When estimating relationships amongst continuous numerical traits, multivariate regression models were performed. A level of *p* < 0.05 was deemed statistically significant. Considering the central choroidal thickness and spot measurements of choroidal thickness, relative changes (decrease) were calculated individually. Descriptive statistics were performed for the relative decreases in all study participants’ eyes and by the prevalence of CSC, strengthened by a multifactor analysis aimed at testing the statistical significance of differences between the study groups. To further verify the research concept, ROC curves, using Youden’s method, were plotted for the measured decreases in choroidal thickness. Proposed empirical cut-off points were read from the ROC curves, and an AUC was computed. The statistical verdict was based on the identification of relative decreases in choroidal thickness where AUC > 0.60 and *p* < 0.05. ROC curves for point measurement were compared using the DeLong, DeLong, and Clarke-Pearson algorithm. All the procedures were carried out using Statistica™, release 13.3 (TIBCO Software Inc., Palo Alto, CA, USA).

## 3. Results

CSC eyes showed significantly impaired BCVA compared to controls. The mean logMAR BCVA in the CSC group was 0.32 ± 0.31 (median 0.20; Q1–Q3: 0.05–0.50) versus 0.0 in controls (*p* < 0.0001).

**Figure 3:**
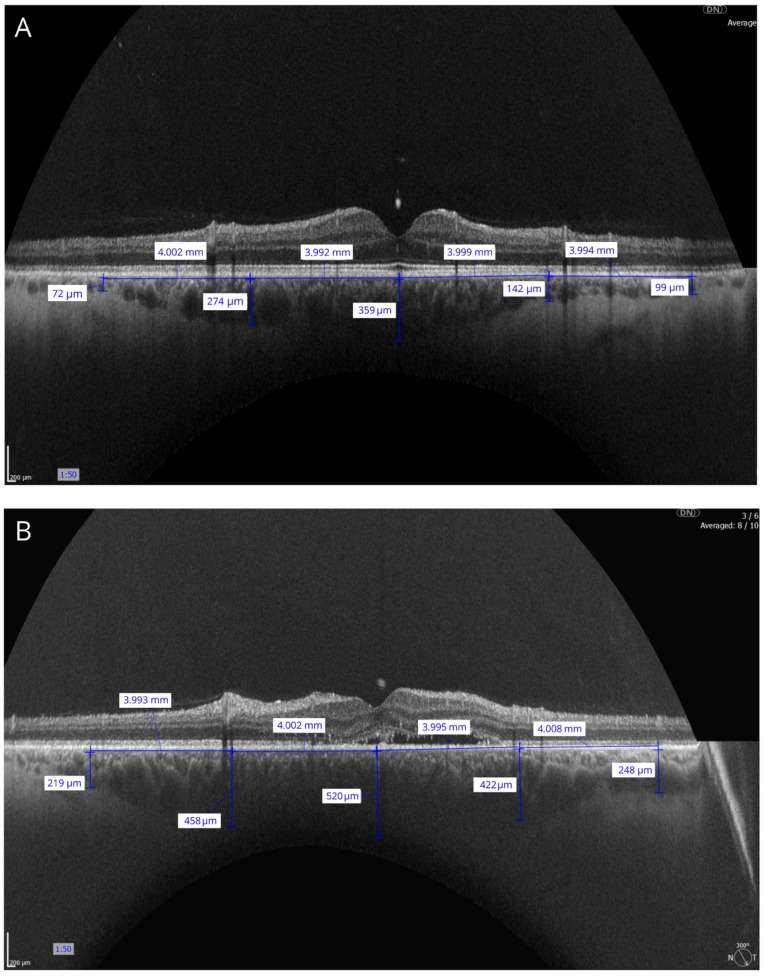
(**A**,**B**). (**A**) Example of choroidal thickness measurements in healthy eyes. (**B**) Example of choroidal thickness measurement in affected eyes.

**Figure 4:**
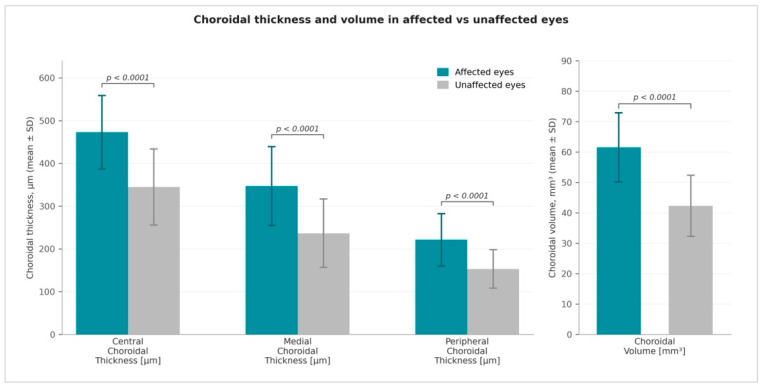
Visualization of the difference in choroidal thickness and volume between the study and control groups.

Choroidal thickness was significantly greater in CSC eyes at all 24 measurement points compared to healthy controls (Supplementary Table S1). Mean CT was elevated subfoveally and at every point on both the 4 mm and 8 mm rings (Table 1). The relative increase in CT in CSC eyes compared to controls was approximately 37% subfoveally, 46% at the mid-periphery, and 44% at the far periphery, indicating that choroidal thickening is diffuse and particularly pronounced in the peripheral zones. Consistent with this finding, mean CV was also significantly higher in CSC patients, with an increase of approximately 45% relative to controls (Table 1). Representative CT measurement maps for an affected and a control eye are shown in Figure 3A,B. Differences in choroidal thickness and volume between affected and unaffected eyes are visualized in Figure 4.

**Figure 5:**
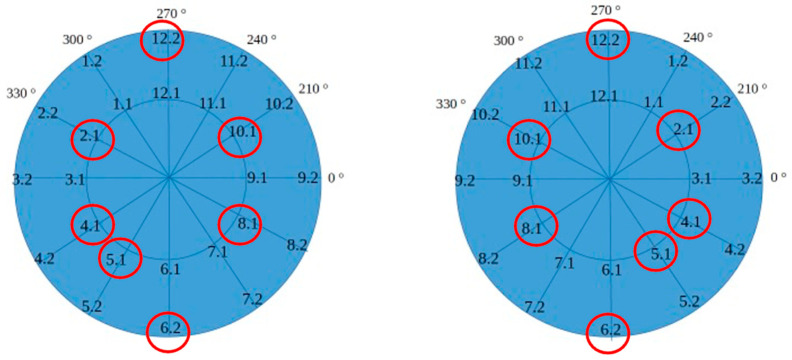
Distribution of significant choroidal thickening in relation to healthy eyes (right eye on the left and left eye on the right—facing the patient) according to ROC curve analysis.

To further characterize the spatial pattern of choroidal thickening, the absolute and relative decrease in CT from the subfoveal reference point towards the periphery was calculated for each measurement location (Supplementary Tables S3 and S4). ROC curve analysis of these relative decreases, using Youden’s method, is presented in Table 2. Modest exploratory discrimination (AUC > 60, *p* < 0.05) was identified at several peripheral measurement points, with the strongest performance at point 8.1 (AUC = 72.4; *p* < 0.0001). Notably, significant points clustered at meridians corresponding to 2/8, 4/10, and 6/12 clock hours, suggesting a non-uniform, sector-dependent pattern of choroidal congestion (Figure 5).

Choroidal volume, derived from the nonlinear model described in the Methods, was significantly greater in CSC eyes than in controls (Table 1). Multivariate logistic regression with standardized explanatory variables and adjusted odds ratios demonstrated that CV had a strong association with CSC, with OR = 2.88; 95% CI: 1.53–5.42 versus OR = 1.04; 95% CI: 1.02–1.07 for central choroidal thickness.

Sectoral differences in choroidal thickness are also reflected in differences in calculated CV in specific sectors, as shown in Table 3. A significant increase in CV is noted for each of the 12 sectors.

Mean relative increases in the CV in specific sectors generally correspond to choroidal thickness increase at specific points (Table 4).

### Correlations

No significant correlation was found between choroidal parameters (CT or CV) and age or BCVA in the CSC group (Supplementary Tables S4 and S5). However, significant positive correlations were identified between all choroidal parameters and disease duration counted from the first episode (Table 5), with Spearman’s rho values ranging from 0.35 to 0.41 across measurement zones and CV (all *p* ≤ 0.0004).

## 4. Discussion

The results of our study confirm the pachychoroid anatomy in eyes affected by CSC. Using UWF SD-OCT, we demonstrated significantly greater CT at all 24 measurement points in CSC patients compared to healthy controls, extending from the subfoveal region to the far periphery. Crucially, the relative increase in CT was more pronounced at the peripheral zones (+44–46%) than subfoveally (+37%), indicating that diffuse rather than focal choroidal expansion characterizes the disease. These findings may be consistent with the venous overload choroidopathy hypothesis proposed by Spaide et al. [4], wherein congestion of the choroidal venous outflow leads to global—not merely central—pachychoroid changes. Nevertheless, such a relationship has to be confirmed by angiographic studies.

The association between increased subfoveal CT and CSC has been firmly established in the literature. Jirarattanasopa et al. were among the first to systematically document macular choroidal thickening in CSC using swept-source OCT and to correlate CT with angiographic abnormalities [10]. This was subsequently confirmed at scale by Chen et al. in a meta-analysis of 12 studies (1108 eyes), demonstrating a weighted mean difference in subfoveal CT of approximately 145 µm between CSC and control eyes [6]. Our results—showing a mean subfoveal difference of approximately 128 µm (472.6 vs. 344.8 µm)—are consistent with this pooled estimate and support the robustness of subfoveal CT as a diagnostic biomarker across different imaging devices and populations.

Data on peripheral choroidal changes in CSC remain less consistent across the literature, partly because most OCT devices are limited to the central macular area. Among studies employing wide-field or UWF imaging, Funatsu et al. [11] and Meng et al. [12] demonstrated increased CT throughout the choroid, including peripheral regions, whereas Nishihara et al. [13] and Izumi et al. [14], also using wide-field OCT, did not confirm peripheral differences—possibly due to smaller sample sizes or patient selection. Our cohort of 50 CSC and 56 control eyes adds meaningful statistical weight to the debate, providing evidence that peripheral choroidal thickening is a genuine and quantifiable feature of CSC. The finding that relative thickening was greater peripherally than subfoveally may reflect uneven distribution of venous congestion across individual vortex vein drainage territories—a hypothesis that future ICGA-correlated studies could directly address.

The distribution of significant choroidal thickening was not uniform across the posterior pole, as illustrated by the ROC curve analysis. Significant discriminative CT points (AUC > 0.60, *p* < 0.05) clustered at specific meridians corresponding to 2/8, 4/10, and 6/12 clock hours. This asymmetric pattern is consistent with reports of the focal nature of pachychoroid vascular abnormalities underlying CSC, in keeping with current concepts of the pachychoroid disease spectrum [15,16]. The clustering of discriminative zones at specific meridians may correspond to the drainage territories of individual dilated choroidal veins, as suggested by Cheung [15], though this interpretation requires validation against ICGA findings that were not available in the present retrospective study.

Choroidal volume extends the descriptive power of single-point CT measurements by capturing the three-dimensional extent of choroidal expansion. Despite its potential clinical relevance, CV has been incorporated into CSC research in only a limited number of studies. Pertl et al. demonstrated, in a retrospective series of 27 untreated CSC eyes, that increases in subretinal fluid are coupled with increases in CV—pointing to a dynamic relationship between choroidal congestion and fluid accumulation [17]. Zeng et al. subsequently reported elevated CV and choriocapillaris density across multiple sectors using UWF swept-source OCT-angiography [18], while Maruko et al. found increased CV in the central but not peripheral areas in CSC [19]. The recent deep-learning study by Valsecchi et al. confirmed higher volumetric choroidal parameters across all sectors [20]. Our study adds to this body of evidence by demonstrating a 45% increase in mean CV in CSC eyes compared to controls and—crucially—showing that CV carries a higher odds ratio for CSC occurrence than central CT alone (OR = 1.16 vs. 1.03). This suggests that volumetric assessment captures global disease burden more faithfully than any single-point measurement.

A further contribution of our study is the positive correlation between both CT and CV and disease duration (Spearman rho 0.35–0.41; *p* ≤ 0.0004). Long-lasting, complex cases tend to have higher values of choroidal thickness and volume. The absence of a significant correlation between choroidal parameters and BCVA is consistent with earlier observations that visual function in CSC is determined primarily by photoreceptor integrity rather than by CT per se—as demonstrated by Piccolino et al. for outer photoreceptor layer atrophy [21], Yoneyama et al. for ellipsoid zone continuity in resolved CSC [22], and Nagase et al. for EZ integrity in non-resolving disease [23]. CT and CV should therefore be regarded as markers of disease chronicity and choroidal burden, complementary to—rather than predictive of—visual acuity.

The clinical relevance of choroidal biomarkers extends naturally to treatment monitoring. Effective therapies for CSC—in particular, half-dose or half-fluence photodynamic therapy—are known to reduce CT as part of their mechanism of action. Despite this mechanistic basis, the comparative effectiveness of available treatments remains uncertain. The 2025 Cochrane network meta-analysis by Lange et al.—the comprehensive synthesis of CSC interventions to date—concluded that different treatment options, especially low-dose PDT, mineralocorticoid receptor antagonists, or supplements, may be effective, but the quality of evidence remains limited [24]. Moreover, subthreshold micropulse laser—so far regarded as a viable treatment for CSC primarily through its action on RPE function—should also be evaluated with respect to possible choroidal remodeling [25,26,27]. In this context, objective and reproducible biomarkers such as CV and peripheral CT could serve as sensitive endpoints in future clinical trials, enabling quantification of treatment response across the full choroidal volume rather than the subfoveal point alone.

In the present study, we concentrated on structural changes in the choroid based on the UWF OCT exam. Nevertheless, the employment of devices evaluating retinal and choroidal vascularity could provide additional information on the analyzed subject. Choroidal hemodynamic changes are increasingly recognized as fundamental to CSC pathogenesis. Xu et al. recently demonstrated significant alterations in choroidal blood flow in CSC patients, suggesting that abnormal vascular dynamics may precede or drive structural changes [16]. Our structural findings on peripheral CT and CV complement this hemodynamic perspective: if vascular congestion is diffuse, metrics capturing total choroidal volume are likely to reflect disease burden more faithfully than subfoveal CT alone. Integration of functional blood flow imaging and structural volumetric OCT measurements may therefore represent a promising direction for future comprehensive CSC characterization. Hence, future studies combining UWF structural OCT with standardized wide-field OCTA reporting and quantitative choroidal-flow metrics may help determine whether regional thickness and volume changes correspond to true sectoral perfusion abnormalities” [15,16,28,29].

In summary, our study demonstrates that UWF SD-OCT provides a clinically meaningful and comprehensive assessment of choroidal architecture in CSC, extending well beyond conventional subfoveal measurements. Choroidal volume emerges as a sensitive and discriminative biomarker that correlates with disease duration and outperforms central CT in association with CSC status. These findings support the incorporation of CV and peripheral CT measurements into routine clinical evaluation and future research protocols for CSC.

### Study Limitations

The retrospective design and modest sample size may limit the generalizability of the findings. The absence of ICGA data precluded direct correlation of the focal choroidal thickening pattern—particularly the meridional clustering identified on ROC analysis—with underlying vascular abnormalities; such correlation is planned for subsequent studies. Moreover, swept-source OCT or UWF OCT angiography—none of which was applied in our study—could have provided greater detail on choroidal architecture, particularly the vascular choroidal layers.

Choroidal volume was estimated using a mathematical model rather than automated volumetric segmentation, although the consistent application of the same method to both groups ensures a valid between-group comparison. Manual CT measurements, while standardized, carry inherent inter-observer variability. In this context, advanced enhanced depth imaging—preferably obtained with a swept-source device, which was not available in the present study—could have provided superior visualization of the scleral interface and improved the reliability of manual measurements.

Our analysis did not include biometric factors such as axial length or refractive error, nor systemic aspects such as comorbidities or drug intake. These factors should be addressed in future studies.

It also has to be noted that disease duration was estimated on the basis of medical history.

## 5. Conclusions

UWF SD-OCT confirms diffuse pachychoroid thickening in CSC, with relative peripheral increases exceeding those observed subfoveally, consistent with global venous overload choroidopathy. Choroidal volume was increased by approximately 45% in CSC eyes and carried a higher odds ratio for CSC occurrence than central CT alone (OR = 2.88 vs. 1.04), positioning CV as a comprehensive biomarker of disease burden. Both CT and CV correlated positively with disease duration but not with BCVA, indicating that these parameters reflect structural choroidal burden independently of visual function. Incorporation of UWF-derived CV and peripheral CT into clinical evaluation and future trial protocols may improve prognostic stratification and treatment monitoring in CSC.

## Figures and Tables

**Table 1:** Baseline characteristics of the study participants’ eyes by group (*n* = 106).

	Affected Eyes			Unaffected Eyes			*p* Value
	*n* (%)						
No. of eyes	50 (47.2)			56 (52.8)			n/a
Gender:							
● Female	6 (14.6)			8 (25.0)			0.2676
● Male	35 (85.4)			24 (75.0)			
	M (SD), Me (Q_1_–Q_3_), SE (95% CI)						
Age [y]	47.6 (9.6)	47.5 (42–53)	1.4 (44.9–50.4)	44.9 (10.9)	46.0 (39–51)	1.4 (41.9–47.8)	0.1602
Disease duration [m]	47.7 (48.5)	35 (15–48)	6.9 (33.9–61.4)	n/a	n/a	n/a	n/a
BCVA [logMAR]	0.32 (0.31)	0.20 (0.05–0.50)	0.04 (0.23–0.41)	0.00 (0.00)	0.00	0.00	<0.0001
Central Choroidal Thickness [µm]	472.6 (8.6)	484.5 (435–513)	8.6 (455.4–489.9)	344.8 (8.9)	351.0 (305–389)	8.9 (327.0–362.6)	<0.0001
Medial Choroidal Thickness [µm]	346.8 (9.2)	361.7 (326–394)	9.2 (328.3–365.2)	236.6 (8.0)	224.5 (192–288)	8.0 (220.6–252.6)	<0.0001
Peripheral Choroidal Thickness [µm]	221.1 (6.1)	232.2 (199–248)	6.1 (208.8–233.5)	153.1 (4.5)	152.6 (129–174)	4.5 (144.0–162.1)	<0.0001
Choroidal Volume [mm^3^]	61.47 (11.37)	64.42 (56.74–69.78)	1.61 (58.23–64.70)	42.29 (10.02)	40.73 (34.37–50.50)	1.34 (39.60–44.97)	<0.0001

*n*—number, %—percentage, M—mean, SD—standard deviation, SE—standard error, Me—median.

**Table 2:** ROC curve analysis using Youden’s method for the mean relative decreases in the spot measurements from the central choroidal thickness [%] in the study participants’ eyes (*n* = 106).

Choroidal Thicknesses	Cut-Off Point	AUC		False-Positive Rate	*p* Value	
		Value	95% CI			
**Central**						<0.0001
**9.2**	40.8	49.8	38.7–60.9	39.0	0.9699	
**3.1**	33.9	53.7	42.6–64.7	35.9	0.5138	
**3.2**	39.8	40.4	29.5–51.3	39.6	0.0851	
**10.2**	57.4	51.9	40.7–63.0	38.2	0.7437	
**10.1**	33.0	63.5	52.8–74.2	32.9	0.0133	
**4.1**	30.8	65.8	55.3–76.3	33.3	0.0031	
**4.2**	57.6	60.2	49.2–71.2	35.7	0.0697	
**11.2**	63.2	53.5	42.4–64.5	35.7	0.5381	
**11.1**	33.0	59.6	48.7–70.5	34.8	0.0850	
**5.1**	35.1	62.0	51.3–72.6	33.8	0.0277	
**5.2**	58.8	55.2	44.2–66.2	36.0	0.3511	
**12.2**	62.1	68.1	57.9–78.3	33.8	0.0005	
**12.1**	29.7	44.3	33.1–55.5	34.1	0.3200	
**6.1**	34.5	52.6	41.5–63.6	34.9	0.6505	
**6.2**	61.6	67.4	57.0–77.8	33.3	0.0011	
**7.2**	64.8	52.6	41.6–63.7	40.0	0.6400	
**7.1**	21.0	58.4	47.3–69.4	34.6	0.1384	
**1.1**	39.6	54.5	43.4–65.5	35.5	0.4272	
**1.2**	56.1	52.4	41.3–63.5	39.1	0.6735	
**8.2**	55.8	58.0	47.1–68.8	37.1	0.1514	
**8.1**	32.8	72.4	62.3–82.5	33.8	<0.0001	
**2.1**	29.3	66.5	56.0–77.0	34.1	0.0021	
**2.2**	66.1	54.3	43.3–65.3	37.7	0.4455	

ROC—Receiver Operating Characteristic, AUC—area under the ROC curve, CI—confidence interval. The penultimate right column contains *p* values for the individual point measurements and their relevant AUC. The rightmost column in the table displays the *p* value for the multiple ROC curve comparisons by using the DeLong, DeLong, and Clarke-Pearson algorithm.

**Table 3:** Values of choroidal volume in specific sectors in the study and control groups.

	Study Group (*n* = 50)		Control Group (*n* = 56)		
Sector †	M (SD)	Me (Q1–Q3)	M (SD)	Me (Q1–Q3)	*p* Value *
12 o’clock	5.63 (0.99)	5.90 (5.12–6.20)	3.96 (1.05)	4.14 (3.04–4.76)	<0.001
1 o’clock	5.52 (1.13)	5.66 (5.12–6.09)	4.05 (1.14)	3.75 (3.31–4.81)	<0.001
2 o’clock	5.23 (1.14)	5.41 (4.53–6.03)	3.48 (1.01)	3.32 (2.69–4.08)	<0.001
3 o’clock	5.41 (1.10)	5.48 (4.44–6.19)	3.91 (1.02)	3.96 (3.06–4.45)	<0.001
4 o’clock	5.49 (1.08)	5.67 (5.08–6.15)	3.48 (1.08)	3.26 (2.53–4.18)	<0.001
5 o’clock	5.79 (1.11)	6.05 (5.08–6.52)	3.98 (1.06)	3.94 (3.24–4.81)	<0.001
6 o’clock	4.85 (1.13)	5.10 (4.18–5.77)	3.35 (0.91)	3.42 (2.59–4.03)	<0.001
7 o’clock	4.70 (1.24)	4.92 (3.96–5.38)	3.18 (0.97)	3.04 (2.50–3.95)	<0.001
8 o’clock	4.49 (1.19)	4.65 (4.00–5.29)	3.00 (0.86)	3.04 (2.43–3.44)	<0.001
9 o’clock	5.08 (1.10)	5.48 (4.24–5.91)	3.78 (0.94)	3.74 (3.06–4.37)	<0.001
10 o’clock	4.43 (1.01)	4.75 (4.04–5.10)	3.01 (0.87)	3.03 (2.35–3.58)	<0.001
11 o’clock	4.84 (1.24)	4.91 (4.27–5.68)	3.11 (0.94)	3.23 (2.24–3.78)	<0.001
Total	61.47 (11.37)	64.42 (56.74–69.78)	42.29 (10.02)	40.73 (34.37–50.50)	<0.001

* Mann–Whitney U test (two-tailed). Values in mm^3^. M = mean; SD = standard deviation; Me = median; Q1–Q3 = first and third quartiles. † Clock-hour positions are referenced as viewed by the examiner facing the patient. For the right eye (OD), sectors are counted counterclockwise; for the left eye (OS), clockwise. The 9 o’clock sector corresponds to the nasal meridian in both eyes.

**Table 4:** Relative increase in choroidal volume for specific sectors in affected eyes in reference to control eyes.

Sector	Study Group (*n* = 50)	Control Group (*n* = 56)	Relative Increase	Rank
(Clock Position)	M (SD), mm^3^	M (SD), mm^3^	(%)	
4 o’clock	5.49 (1.08)	3.48 (1.08)	57.9%	1
11 o’clock	4.84 (1.24)	3.11 (0.94)	55.7%	2
2 o’clock	5.23 (1.14)	3.48 (1.01)	50.1%	3
8 o’clock	4.49 (1.19)	3.00 (0.86)	49.6%	4
7 o’clock	4.70 (1.24)	3.18 (0.97)	47.7%	5
10 o’clock	4.43 (1.01)	3.01 (0.87)	47.3%	6
5 o’clock	5.79 (1.11)	3.98 (1.06)	45.5%	7
6 o’clock	4.85 (1.13)	3.35 (0.91)	44.9%	8
12 o’clock	5.63 (0.99)	3.96 (1.05)	42.3%	9
3 o’clock	5.41 (1.10)	3.91 (1.02)	38.4%	10
1 o’clock	5.52 (1.13)	4.05 (1.14)	36.2%	11
9 o’clock	5.08 (1.10)	3.78 (0.94)	34.4%	12
Total	61.47 (11.37)	42.29 (10.02)	45.4%	

Sectors sorted by relative increase (highest to lowest). Clock-hour positions referenced as viewed by the examiner. For OD, sectors counted counterclockwise; for OS, clockwise. The 9 o’clock sector corresponds to the nasal meridian in both eyes. Relative increase = (Study mean − Control mean)/Control mean × 100%.

**Table 5:** Spearman correlation of choroidal thickness and volume with CSC duration for the affected eyes: Spearman *rho* coefficients.

Parameter	R	*p*
Central choroidal thickness	0.36	0.0002
Medial choroidal thickness	0.41	<0.0001
Peripheral choroidal thickness	0.35	0.0004
Choroidal volume	0.36	0.0002

R—rho Spearman’s coefficient.

## Data Availability

The original contributions presented in this study are included in the article and Supplementary Material.

## Supplementary Materials

The following supporting information can be downloaded at: https://www.mdpi.com/article/10.3390/diagnostics16131982/s1. Supplementary Material Table S1. Spot measurements of choroidal thickness [µm] in the study participants’ eyes by group (*n* = 106); Supplementary Material Table S2. Mean absolute differences in the spot measurements from the central choroidal thickness [µm] in the study participants’ eyes by group (*n* = 106); Supplementary Material Table S3. Mean relative decreases in the spot measurements from the central choroidal thickness [%] in the study participants’ eyes by group (*n* = 106); Supplementary Material Table S4. Spearman correlation of choroidal thickness and volume with age for the affected eyes, *rho* coefficients; Supplementary Material Table S5. Spearman correlation of choroidal thickness and volume with BCVA for the affected eyes, *rho* coefficients.
