# Supplementary material for: Ultra-Wide-Field Optical Coherence Tomography Assessment of Choroidal Parameters in Central Serous Chorioretinopathy
Source: Diagnostics (Basel). 2026 Jun 25;16(13):1982. doi: 10.3390/diagnostics16131982 (PMC13359502; doi:10.3390/diagnostics16131982)
Supplement: Supplementary file 1 [file diagnostics-16-01982-s001.zip › diagnostics-4313019-supplementary.pdf]

Supplementary Material Table S1. Spot measurements of choroidal thickness [μm] in the study participants' eyes by group (n = 106)

Choroidal thicknesses	Affected eyes		Unaffected eyes		*P value*	
	M, SD					
Central	472.6	60.6	344.8	66.4	<0.0001	<0.0001
9.2	249.9	74.8	184.5	58.4	<0.0001	<0.0001
3.1	340.8	75.0	245.1	74.2	<0.0001	<0.0001
3.2	224.0	65.1	172.9	39.6	<0.0001	<0.0001
10.2	213.2	59.2	156.8	56.0	<0.0001	<0.0001
10.1	333.0	73.1	220.2	66.8	<0.0001	<0.0001
4.1	332.8	73.6	221.6	65.8	<0.0001	<0.0001
4.2	232.5	65.6	156.5	56.6	<0.0001	<0.0001
11.2	232.7	60.3	161.5	50.9	<0.0001	<0.0001
11.1	351.0	81.5	238.6	72.8	<0.0001	<0.0001
5.1	254.7	77.0	240.1	71.5	<0.0001	<0.0001
5.2	233.4	93.7	161.5	49.4	<0.0001	<0.0001
12.2	227.6	65.2	136.3	50.8	<0.0001	<0.0001
12.1	390.5	62.9	286.2	77.2	<0.0001	<0.0001
6.1	346.3	80.7	244.9	70.1	<0.0001	<0.0001
6.2	175.9	50.2	109.6	31.5	<0.0001	<0.0001
7.2	225.2	91.0	154.3	66.6	<0.0001	<0.0001
7.1	355.1	88.6	243.3	79.1	<0.0001	<0.0001
1.1	350.7	82.4	244.3	85.7	<0.0001	<0.0001
1.2	218.4	77.2	154.8	66.3	<0.0001	<0.0001
8.2	218.9	72.2	145.1	51.2	<0.0001	<0.0001
8.1	335.1	90.1	207.5	69.8	<0.0001	<0.0001
2.1	324.6	83.5	211.3	64.9	<0.0001	<0.0001
2.2	202.1	61.1	142.94	51.1	<0.0001	<0.0001

M – mean, SD – standard deviation. The penultimate right column contains P values for the individual point measurement comparisons. The rightmost column in the table displays the FDR-corrected P value. FDR – false discovery rate

Supplementary Material Table S2. Mean absolute differences in the spot measurements from the central choroidal thickness [μm] in the study participants' eyes by group (n = 106)

Choroidal thicknesses	Affected eyes		Unaffected eyes		P value	
	M, SE					
Central						
9.2	222.7	9.4	160.3	6.4	<0.0001	<0.0001
3.1	131.8	6.9	99.7	6.8	0.0030	0.0032
3.2	248.7	8.2	171.9	7.6	<0.0001	<0.0001
10.2	259.4	8.4	188.0	8.6	<0.0001	<0.0001
10.1	139.6	7.2	124.6	6.9	0.0904	0.1238
4.1	139.8	8.7	123.2	6.3	0.1657	0.2135
4.2	240.2	9.7	188.3	5.5	0.0005	0.0004
11.2	239.9	7.0	183.2	8.0	<0.0001	<0.0001
11.1	121.7	6.6	106.2	7.4	0.1535	0.1990
5.1	117.9	8.1	104.7	6.6	0.1880	0.2135
5.2	239.2	8.0	183.3	7.2	<0.0001	<0.0001
12.2	245.0	7.3	208.4	8.4	0.0007	0.0006
12.1	82.2	5.3	58.6	6.0	0.0006	0.0006
6.1	126.4	7.4	99.9	7.2	0.0050	0.0059
6.2	296.7	6.9	235.2	8.2	<0.0001	<0.0001
7.2	247.7	10.7	190.5	10.5	0.0003	0.0003
7.1	117.5	8.3	101.5	8.6	0.1923	0.2135
1.1	122.0	8.5	100.5	7.9	0.0655	0.0922
1.2	254.3	11.9	190.0	8.8	0.0001	<0.0001
8.2	253.8	8.6	199.7	8.0	<0.0001	<0.0001
8.1	137.6	8.2	137.3	7.3	0.8173	0.8176
2.1	148.0	9.0	133.5	6.9	0.6215	0.6598
2.2	270.6	9.3	201.8	7.9	<0.0001	<0.0001

M – mean, SD – standard deviation. The penultimate right column contains P values for the individual point measurement comparisons. The rightmost column in the table displays the FDR-corrected P value. FDR – false discovery rate.)

Supplementary Material Table S3. Mean relative decreases in the spot measurements from the central choroidal thickness [%] in the study participants' eyes by group (n = 106)

Choroidal thicknesses	Affected eyes		Unaffected eyes		P value	
	M, SE					
Central						
9.2	47.4	1.9	46.8	1.6	0.9580	0.9575
3.1	28.3	1.5	29.1	2.1	0.3560	0.4845
3.2	52.9	1.6	48.9	1.5	0.0159	0.0368
10.2	55.0	1.5	54.2	1.9	0.6416	0.6745
10.1	29.9	1.7	36.3	1.9	0.0007	0.0022
4.1	29.7	1.9	36.1	1.6	<0.0001	0.0002
4.2	50.8	1.8	54.3	1.9	0.0105	0.0323
11.2	51.1	1.4	52.7	1.7	0.3848	0.4934
11.1	26.4	1.6	30.9	2.0	0.0162	0.0368
5.1	25.3	1.7	30.8	1.8	0.0027	0.0071
5.2	50.8	1.5	53.1	1.5	0.1891	0.3118
12.2	52.3	1.6	60.2	1.7	<0.0001	<0.0001
12.1	17.5	1.1	17.4	1.8	0.1541	0.2875
6.1	27.3	1.7	29.1	2.0	0.5219	0.6020
6.2	63.1	1.2	67.6	1.2	<0.0001	<0.0001
7.2	53.0	2.4	54.8	2.4	0.5075	0.6020
7.1	25.5	2.0	29.8	2.4	0.0359	0.0792
1.1	26.2	1.9	29.7	2.4	0.2626	0.4048
1.2	53.7	2.2	55.2	2.0	0.5485	0.6056
8.2	54.1	1.8	57.9	1.7	0.0455	0.0848
8.1	29.9	2.0	40.4	1.9	<0.0001	<0.0001
2.1	31.8	2.0	38.5	2.1	<0.0001	0.0001
2.2	27.2	1.7	58.2	1.8	0.2822	04068

M – mean, SD – standard deviation. The penultimate right column contains P values for the individual point measurement comparisons. The rightmost column in the table displays the FDR-corrected P value. FDR – false discovery rate

Supplementary Material Table S4. Spearman correlation of choroidal thickness and volume with age for the affected eyes, *rho* coefficients:

Parameter	R	p
Central choroidal thickness	-0.19	0.0602
Medial choroidal thickness	-0.13	0.0602
Peripheral choroidal thickness	-0.06	0.5234
Choroidal volume	-0.10	0.3195

Supplementary Material Table S5 Spearman correlation of choroidal thickness and volume with BCVA for the affected eyes, *rho* coefficients:

Parameter	R	p
Central choroidal thickness	0.16	0.1155
Medial choroidal thickness	0.15	0.1413
Peripheral choroidal thickness	0.05	0.6040
Choroidal volume	0.12	0.2306
